# Acute post-cardiopulmonary bypass left atrial thrombosis after mitral valvuloplasty and left atrial thrombectomy

**DOI:** 10.1186/1749-8090-7-5

**Published:** 2012-01-11

**Authors:** Dong-Hyup Lee, Tae-Eun Jung, Sang-Jin Park

**Affiliations:** 1 Department of Thoracic and Cardiovascular Surgery, College of Medicine, Yeungnam University, Daegu, Korea; 2 Department of Anesthesia and Pain Medicine, College of Medicine, Yeungnam University, Daegu, Korea

**Keywords:** mitral valvuloplasty, acute thrombosis, cardiopulmonary bypass

## Abstract

A patient with mitral stenosis and multiple left atrial thrombi underwent valvuloplasty and thrombectomy. While closing the sternum after completing the cardiopulmonary bypass, a new left atrial thrombus was detected by transesophageal echocardiography. We used heparin for the prevention of new thrombus formation and closed the wound after meticulous bleeding control. Three months later, there was no residual thrombus in the left atrium according to the echocardiographic study.

## Background

Acute left atrial thrombosis immediately after cardiopulmonary bypass is rare, but it is a serious and sometimes fatal complication during cardiac surgery [1,2]. The factors associates with acute thrombosis during cardiac surgery include the use of aprotinin, a long pump time, the use of heparin and protamine, and transfusion of blood components. The other factors associates with acute thrombosis include congenital antithrombin III deficiency, acquired protein C and protein S deficiency, antiphospholipid antibody syndrome and hyperhomocysteinemia [3-5]. Each of these factors may cause this complication by itself or by acting in combination [6,7].

We report a case of new left atrial thrombus formation immediately after cardiopulmonary bypass in patient with mitral valvuloplasty and left atrial thrombectomy.

## Case presentation

A 72 years old male patient was hospitalized after a month of heart palpitations. One month previously, the patient complained of slight chest discomfort and palpitations while climbing stairs. The patient received medications at that time, but they did not help relieve the symptoms. The patient had been previously diagnosed with atrial fibrillation 15 years ago, and he had not been treated for it with any medication, including anticoagulants.

On admission to the hospital, a mid-diastolic rumbling murmur was heard (Grade III/VI) at the apex of the chest on the physical examination. A standard chest PA showed an increase of the pulmonary vascular markings and enlargement of the left atrium. The echocardiography showed a mitral valve orifice of 0.83 cm^2^, a mean diastolic pressure gradient of 10 mmHg and a left atrial volume index of 96 mm^3^/m^2^. In addition, there were trivial amounts of mitral regurgitation and multiple variably sized thrombi, including an 8 × 5 cm thrombus, in the septum, posterior wall and entrance of the left auricle.

**Figure 1:**
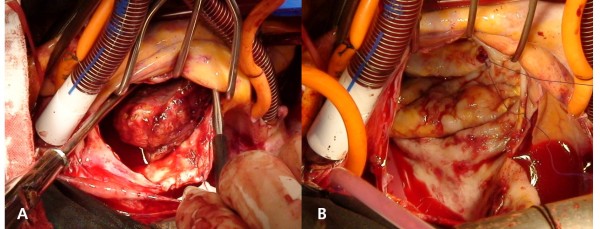
**A) Large thrombus in the left atrium**. B) The surface of the left atrium was very rough even after the removal of thrombi.

**Figure 2:**
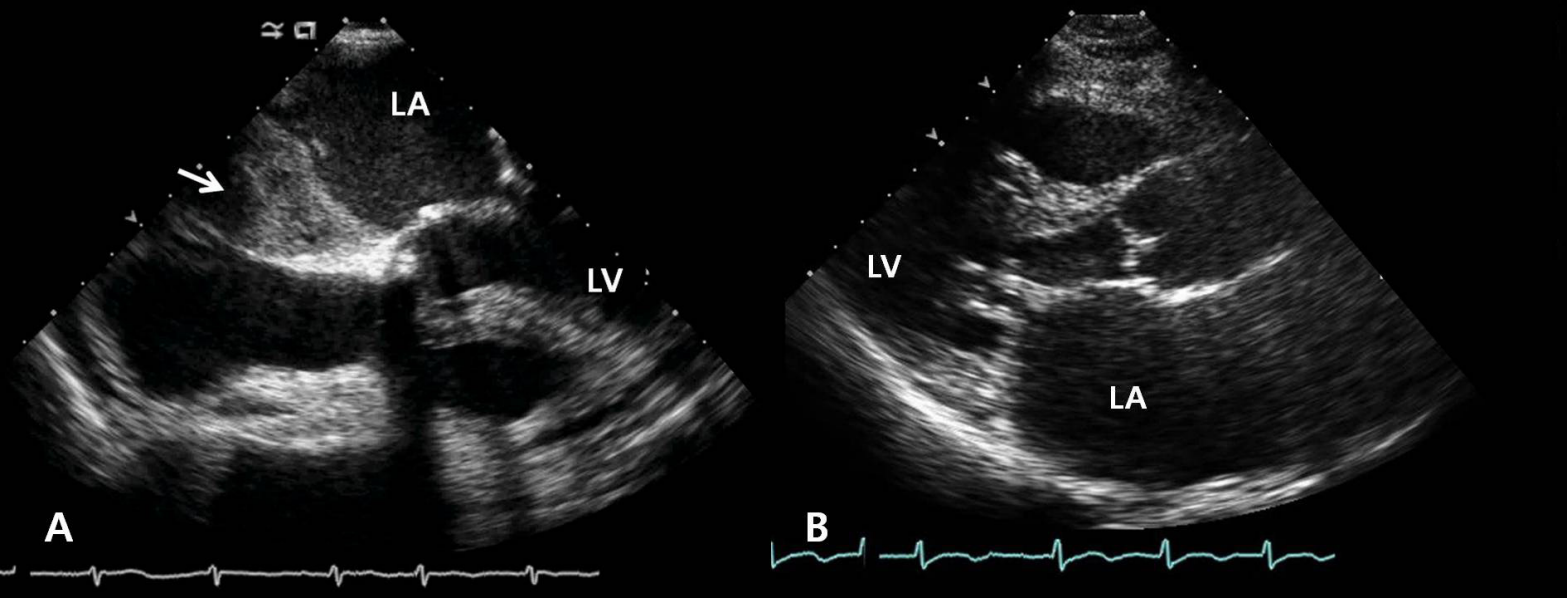
**A) Transesophageal echocardiogram shows new thrombus formation in the left atrium (arrow)**. B) Left atrial thrombus was not seen in transthoracic echocardiogram (postoperative 3 months). LA: left atrium; LV: left ventricle.

Surgery was performed under general anesthesia with superior and inferior vena cava and aortic cannulation. After the procedure, antegrade and retrograde cold blood cardioplegia was used for myocardial protection. The left atrium was opened through the Waterston's groove to remove the multiple thrombi; however, the atrial surface was very rough even after removing the thrombi (Figure 1). Resection of the left atrial auricle, mitral valvuloplasty and partial left atrial volume reduction were performed. During the cardiopulmonary bypass, the ACT remained greater than 450 seconds and there was no problem during weaning from the cardiopulmonary bypass. After protamine was injected, the ACT was 130 seconds. The platelet count was 45,000/mm^3 ^during sternal closure; five units of platelets were then transfused. After this, no mitral regurgitation was observed by trans-esophageal echocardiogram, but a new thrombus was detected at the left atrium (Figure 2). Injection of 0.5 mg of heparin/kg was done to maintain the ACT at greater than 250 seconds and to block the formation of new thrombi. The developing thrombus did not affect the blood flow through the mitral valve; the sternum was closed and the surgery completed.

On that day, the patient received constant doses of heparin until postoperative day 1, and then the medication was changed to Coumadin for further anticoagulation. To determine the cause of thrombus formation, coagulation studies such as hyperhomocysteinemia and heparin-induced thrombocytopenia were performed using a blood sample from surgery. Yet we could not find a definite cause of the new thrombus formation on the hematologic studies. The patient gradually recovered with no special problems. The postoperative echocardiogram on day 10 showed a mitral valve area of 1.77 cm^2 ^and a mean diastolic pressure gradient of 5.1 mmHg. While some signs of thrombus formation at the left atrium were noted during early follow-up, by three months post-surgery none were detected on the follow up echocardiogram (Figure 2).

## Discussion

There were many possible factors which cause thrombosis after cardiopulmonary bypass. As a surgical factors, the use of aprotinin, a long pump time, the use of heparin and protamine, and a transfusion of blood components. The other factors associates with acute thrombosis include congenital antithrombin III deficiency, acquired protein C and protein S deficiency, antiphospholipid antibody syndrome and hyperhomocysteinemia [3-5]. Each of these factors may cause this complication by itself or by acting in combination [6,7].

Our patient was negative for antithrombin III deficiency and hyperhomocysteinemia. The protein C antigen and protein S antigen were decreased on the antiphospholipid antibody study. But the use of heparin for cardiopulmonary bypass might have affected these antigen levels. The use of heparin might have also contributed to the sudden development of acute thrombus formation. The study for heparin-induced thrombocytopenia was negative. We did not use aprotinin. The cardiopulmonary bypass time was 114 minutes and this was no longer than for the usual mitral valve operation. Besides the hematologic reasons, the factors associated with thrombus formation are a large atrial volume and insufficient relief of mitral stenosis. However, in this case, the postoperative area of the mitral valve orifice was 1.77 m^2^, which did not affect blood flow. Another possibility is that curettage of the left atrium for the multiple thrombi left its large surface very rough, and this might have promoted additional thrombus formation. In addition, the platelet transfusion during surgery might have contributed to the development of an acute thrombus. The thrombus was detected by intraoperative transesophageal echocardiography and this procedure was essential for making an early diagnosis.

For management, we thought that if cardiopulmonary bypass was restarted, the opening of the left atrium to remove the thrombus might have increased the risk for repeat thrombus formation during heparin reversal. Injecting thrombolytic agents has a significant risk for bleeding. We decided to solve this problem with injection of low dose heparin for maintaining the ACT at greater than 250 seconds for prevention of new thrombus formation.

## Conclusions

In patient with acute new left atrial thrombus formation immediately after cardiopulmonary bypass, the possible management is to inject anticoagulants with close observation to prevent further formation of thrombus, if the blood flow through the mitral valve isn't affected and there are no signs of bleeding.

### Consent

Written informed consent was obtained from the patient for publication of this case report and accompanying images. A copy of the written consent is available for review by the Editor-in-Chief of this journal.

## Competing interests

The authors declare that they have no competing interests.

## Authors' contributions

DL and SP wrote the draft of the manuscript and obtained the written consent. TJ performed the literature review and participated in the manuscript writing and helped to the final writing of the paper and gave final approval of the manuscript. All authors have read and approved the final manuscript.
